# An Antiviral Peptide from *Alopecosa nagpag* Spider Targets NS2B–NS3 Protease of Flaviviruses

**DOI:** 10.3390/toxins11100584

**Published:** 2019-10-10

**Authors:** Mengyao Ji, Tengyu Zhu, Meichen Xing, Ning Luan, James Mwangi, Xiuwen Yan, Guoxiang Mo, Mingqiang Rong, Bowen Li, Ren Lai, Lin Jin

**Affiliations:** 1 College of Life Sciences, Nanjing Agricultural University, Nanjing 210095, Jiangsu, China; 2 Key Laboratory of Animal Models and Human Disease Mechanisms of Chinese Academy of Sciences/Key Laboratory of Bioactive Peptides of Yunnan Province, Kunming Institute of Zoology, Chinese Academy of Sciences, Kunming 650223, Yunnan, China; 3 The National & Local Joint Engineering Laboratory of Animal Peptide Drug Development, College of Life Sciences, Hunan Normal University, Changsha 410081, Hunan, China; 4 KIZ-CUHK Joint Laboratory of Bioresources and Molecular Research in Common Diseases, Kunming Institute of Zoology, Chinese Academy of Sciences, Kunming 650223, Yunnan, China; 5 Sino-African Joint Research Center, Kunming Institute of Zoology, Chinese Academy of Sciences, Kunming 650223, Yunnan, China; 6 Institute for Drug Discovery and Development, Chinese Academy of Sciences, Shanghai 201203, China; 7 Center for Biosafety Mega-Science, Chinese Academy of Sciences, No.44, Xiaohongshan, Wuchang District/Huangjin Industrial Park, Zhengdian Street, Jiangxia District, Wuhan 430207, Hubei, China

**Keywords:** *Alopecosa nagpag*, dengue virus, Zika virus, host defense peptide, NS2B–NS3 protease, Flavivirus

## Abstract

Flaviviruses are single-stranded RNA viruses predominantly transmitted by the widely distributed *Aedes* mosquitoes in nature. As important human pathogens, the geographic reach of Flaviviruses and their threats to public health are increasing, but there is currently no approved specific drug for treatment. In recent years, the development of peptide antivirals has gained much attention. Natural host defense peptides which uniquely evolved to protect the hosts have been shown to have antiviral properties. In this study, we firstly collected the venom of the *Alopecosa nagpag* spider from Shangri-La County, Yunnan Province. A defense peptide named Av-LCTX-An1a (Antiviral-Lycotoxin-An1a) was identified from the spider venom, and its anti-dengue serotype-2 virus (DENV2) activity was verified in vitro. Moreover, a real-time fluorescence-based protease inhibition assay showed that An1a functions as a DENV2 NS2B–NS3 protease inhibitor. Furthermore, we also found that An1a restricts zika virus (ZIKV) infection by inhibiting the ZIKV NS2B–NS3 protease. Together, our findings not only demonstrate that An1a might be a candidate for anti-flavivirus drug but also indicate that spider venom is a potential resource library rich in antiviral precursor molecules.

## 1. Introduction

Flaviviruses are important human pathogens predominantly transmitted by the widely distributed *Aedes* mosquitoes in nature. Among them, dengue virus (DENV), Japanese encephalitis (JEV), West Nile (WNV) and Zika virus (ZIKV) are the most well-known. DENV is an emerging global epidemic, and its infection causes various clinical symptoms including dengue fever and dengue hemorrhagic fever [1]. DENV exists as four serotypes (DENV-1, -2, -3, and -4), thus creating challenges in the development of conventional vaccines [1,2]. In the past decade, the epidemic potential of ZIKV likely increased with its emergence in Pacific, Americas and Asia [3,4]. ZIKV can be classified into the African lineage, and the Asian lineage and the latter is the recent epidemic strain [5,6,7]. Recent studies have shown that ZIKV can cause severe neurological symptoms and can establish persistent infection in body fluids. Though safe and effective vaccines for JEV are available, currently, no effective vaccines or antiviral drugs have been approved for DENV and ZIKV.

Though vaccination is the primary strategy for preventing viral infections, antivirals can also aid in prevention of DENV and ZIKV infection of at-risk populations. In recent years, the development of peptide antivirals has gained much attention, and peptides have become another source of antiviral drugs due to their better safety and low-cost, compared to chemical compounds [8,9,10]. For example, two scorpion venom peptides Hp1036 and Hp1239 were reported to inhibit Herpes Simplex Virus type 1 (HSV-1) infection [11]. The spider toxin latarcin has been proven as a potent antiviral against DENV, and its fusion protein Latarcin-PAP1-Thanatin has inhibitory effects against Chikungunya virus [12,13]. Another representative defensin-like antiviral peptide BmKDfsin4 from the scorpion *Mesobuthus martensii* Karsch has also been reported to inhibit hepatitis B virus replication [14]. Such animal venom toxins and host defense peptides (HDPs) have been shown to exert antiviral properties through various targets [15]. Recently, the NS2B–NS3 protease, which plays a pivotal role in flaviviruses replication, has gained more attention as an ideal anti-flavivirus target [16]. Though a structural analysis of the NS2B–NS3 protease has facilitated the development of its inhibitors, peptide inhibitors identified from spider venom are rarely reported [17,18,19,20,21].

As successful and widely distributed ancient arthropods, spiders use their venom to subdue prey and deter predators. Previous studies have implied that spider venom is a resource for novel therapeutic peptides including antivirals [12,22,23]. The *Alopecosa nagpag* spider has been found and described for nearly two decades, but the components of its venom have not been studied [24]. In this study, we isolated and characterized an antiviral peptide from the venom of the *A. nagpag* spider and named it Antiviral-Lycotoxin-An1a (Av-LCTX-An1a) according to the rational nomenclature [25]. The anti DENV2 and ZIKV activity of An1a was verified in vitro. Moreover, we also found that An1a restricts DENV2 and ZIKV infection by inhibiting their NS2B–NS3 protease through a real-time fluorescence-based protease inhibition assay. Herein, this study shows that An1a is an antiviral agent candidate against flavivirus infection.

## 2. Results

### 2.1. Identification of An1a from the Venom of A. nagpag

**Figure 1:**
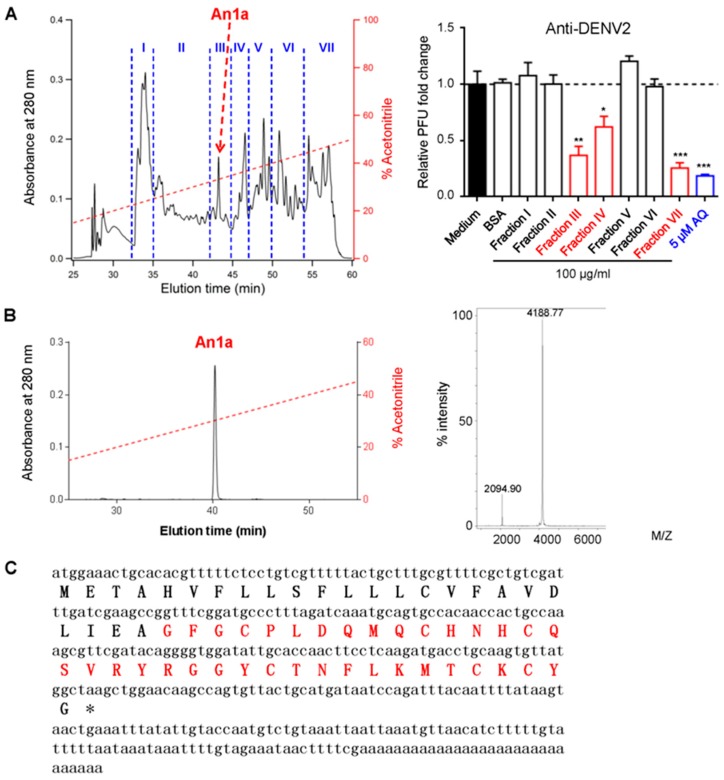
Identification of An1a from the venom of *A. nagpag*. **(A)** Purification of An1a and the antiviral activity determination of peptide fractions. The anti-dengue virus-2 (DENV2) activity of each fraction was tested in Vero cells (right), and fractions with antiviral activities were further purified by using an reverse-phase-(RP)-HPLC column (Unisil C_18_ column, 5 μm particle size and 10 × 250 mm). Elution was performed at a flow rate of 1.5 mL/min with the indicated gradients of acetonitrile in 0.1% (*v*/*v*) TFA (trifluoroacetic acid) in water. Viral titers in the supernatants were determined by plaque assay. Amodiaquine (AQ) was set as the positive control. (**B**) The purity of natural An1a was confirmed to be greater than 99% before further analysis. The average molecular weight of An1a was determined as 4187.77 Da using MALDI-TOF with the LP model (right). (**C**) cDNA encoding the precursor of An1a. The mature peptide is in red and the asterisk indicates stop codon. Data represent three independent experiments and are presented as mean ± SEM. * *p* < 0.05; ** *p* < 0.01; *** *p* < 0.001.

The crude venom was loaded on a Sephadex G-75 (26 × 100 cm; Superfine, Amersham Biosciences) gel filtration column (see the Supplementary Materials for more details). The peptidic fractions with antiviral activities were further purified by using C_18_ reverse-phase-(RP)-HPLC. Elution was performed at a flow rate of 1.5 mL/min with the indicated gradients of acetonitrile in 0.1% (*v*/*v*) trifluoroacetic acid (TFA) in water. As the chromatogram shows in Figure 1A, seven fractions were obtained after RP-HPLC, and their anti-DENV2 activities were screened in Vero cells. An antiviral peptide An1a in Fraction III was purified with an observed molecular weight of 4187.77 Da (Figure 1B). The amino acid sequence of An1a was determined to be GFGCPLDQMQCHNHCQSVRYRGGYCTNFLKMTCKCY by using Edman degradation. The cDNA sequence, and a full-length precursor of An1a was then obtained from the cDNA library of the venom gland of the *A. nagpag* spider by a sequence analysis (Figure 1C and Figure S1). The precursor of An1a was also subjected to the signal peptide prediction tool (SignalP 5.0 Server, http://www.cbs.dtu.dk/services/SignalP/) to verify the signal peptide and mature peptide (Figure 1C).

### 2.2. An1a Inhibits DENV2 Replication

**Figure 2:**
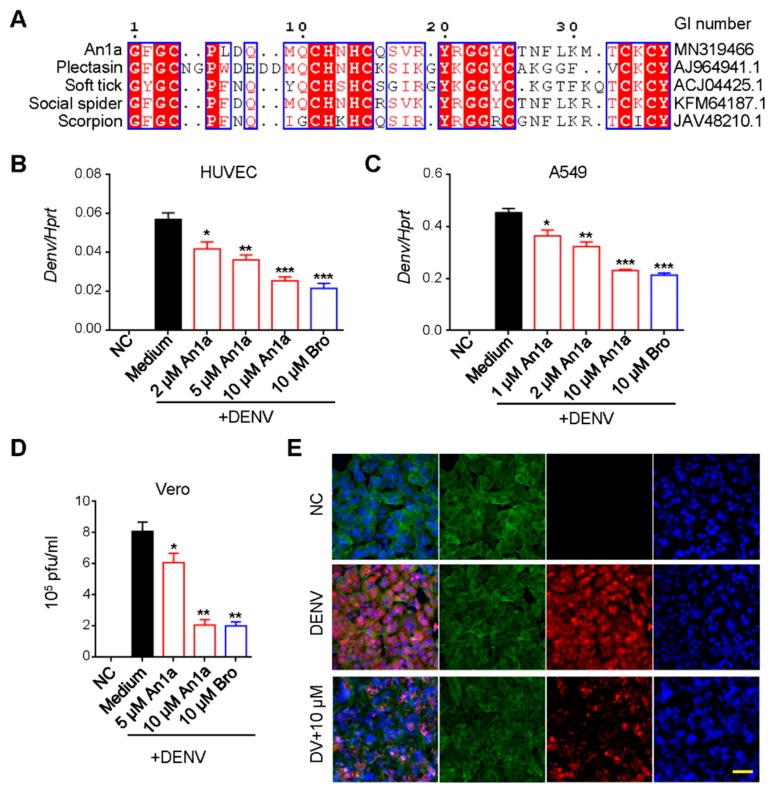
An1a inhibits DENV2 replication. (**A**) Similarity of An1a to selected invertebrate defensins. The figure was generated with the program ESPript. (**B**) qPCR analysis of DENV genes in HUVECs after infection with DENV2 (MOI, 0.5) alone or in combination with An1a or bromocriptine (Bro); results are presented relative to those of human *Hprt*. (**C**) qPCR analysis of DENV genes in A549 cells after infection with DENV2 (MOI, 0.5) alone or in combination with An1a or bromocriptine (Bro); results are presented relative to those of human *Hprt*. (**D**) DENV2 production as detected by plaque forming assay after treatment with An1a in Vero cells. (**E**) Confocal microscopy analysis of DENV2 in Vero cells after treatment with An1a. Scale bar = 20 μM. Data represent three independent experiments in (B)–(E). Data are presented as mean ± SEM. * *p* < 0.05; ** *p* < 0.01; *** *p* < 0.001.

Plectasin, a peptide antibiotic from fungus, has been reported to show significant inhibition activity against DENV replication [26,27]. As shown in Figure 2A, An1a exhibited sequence identities of plectasin and other selected invertebrate defensin-like peptides. Firstly, according to the results, An1a did not show any cytotoxicity and hemolytic activity under 20 μM (Figure S1). To further investigate the antiviral activity of An1a, cells were infected with DENV2 at 1 MOI for 24 h with or without An1a administration. Bromocriptine mesylate, a recently reported antiviral, was chosen as a positive control [21]. We found that An1a significantly inhibited the replication of DENV2 (Figure 2B). We therefore assumed that An1a may also inhibit the virion production of DENV2. As anticipated, the production of DENV2 particles was also restricted by An1a (Figure 2C,D). These results suggested that An1a suppresses DENV2 replication and infectious virus production.

### 2.3. The Inhibitory Activity of An1a on the DENV2 NS2B–NS3 Protease

**Figure 3:**
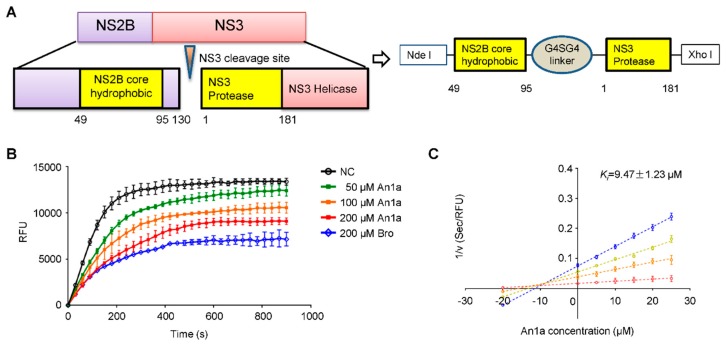
The inhibitory activity of An1a on the DENV2 NS2B–NS3 protease. (**A**) Construction of the recombinant DENV2 NS2B–NS3 protease expression system. NS2B and NS3 were connected by a Gly_4_–Ser–Gly_4_ linker. (**B**) Real-time detection of the inhibitory effect of An1a on the DENV2 NS2B–NS3 protease. (**C**) The Lineweaver–Burk plot shows that An1a is a competitive inhibitor of the DENV2 NS2B–NS3 protease, and the *K_i_* value was determined as 9.47 ± 1.23 μM by the method of Dixon. V is the reaction rate. Data represent three independent experiments.

Current studies have shown that plectasin and its fusion protein significantly inhibit the NS2B–NS3 protease [27,28]. The NS2B–NS3 protease function is essential for flavivirus replication, and it has been regarded as an important antiviral target [16,29]. According to the sequence similarity, An1a may also function as an NS2B–NS3 protease inhibitor. To address this question, we constructed an expression system to obtain a recombinant DENV2 NS2B–NS3 protease which was connected by a Gly_4_–Ser–Gly_4_ linker, as shown in Figure 3A [30]. Real-time detection results suggested that An1a can inhibit the DENV2 NS2B–NS3 protease (Figure 3B). A Dixon analysis showed that An1a is a competitive inhibitor of the DENV2 NS2B–NS3 protease, and the K_i_ value was determined as Ki = 9.47 ± 1.23 μM. The results indicated that An1a may inhibit DENV2 replication and particle production by interacting with the NS2B–NS3 protease.

### 2.4. An1a Also Restricts ZIKV Replication by Inhibiting the NS2B–NS3 Protease

**Figure 4:**
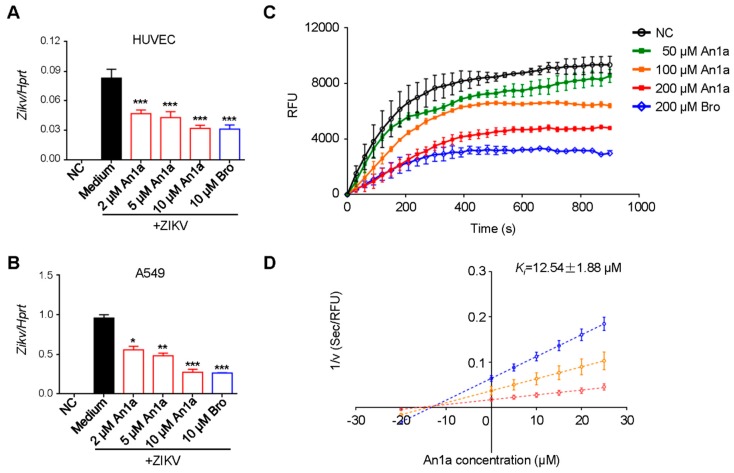
An1a also restricts ZIKV replication by inhibiting its NS2B–NS3 protease. (**A**) qPCR analysis of ZIKV genes in HUVECs and (**B**) A549 cells after infection with ZIKV (MOI, 1) alone or in combination with An1a or bromocriptine (Bro); results are presented relative to those of human *Hprt*. (**C**) Real-time detection of the inhibitory effect of An1a on the ZIKV NS2B–NS3 protease. (**D**) The Lineweaver–Burk plot shows that An1a is a competitive inhibitor of the ZIKV NS2B–NS3 protease, and the *K_i_* value was determined as 12.54 ± 1.88 μM by the method of Dixon. V is the reaction rate. Data represent two independent experiments and are presented as mean ± SEM. * *p* < 0.05; ** *p* < 0.01; *** *p* < 0.001.

The genome of flaviviruses encodes 10 mature viral proteins: C–prM–E–NS1–NS2A–NS2B–NS3–NS4A–NS4B–NS5 [1]. Based on the multiple sequence alignment of the NS2B–NS3 proteases of flaviviruses, most of the residues related to inhibitor binding were conserved (Figure S2). Hence, to explore whether An1a has broad antiviral activity against other flavivirus, we chose ZIKV for further investigation. We found that An1a administration inhibited the replication of ZIKV in HUVEC and A549 cells (Figure 4A,B). Similarly, An1a also showed a remarkable inhibitory effect on the recombinant ZIKV NS2B–NS3 protease which was connected by a Gly_4_–Ser–Gly_4_ linker (Figure 4C). Additionally, the inhibition mechanism of An1a was also determined by a Dixon analysis (Figure 4D). As a competitive inhibitor of the ZIKV NS2B–NS3 protease, the K_i_ value was 12.54 ± 1.88 μM (Figure 4D). Together, our results have shown that An1a may have broad-spectrum anti-flavivirus activity by targeting the NS2B–NS3 protease.

## 3. Discussion

Venom is a kind of formidable biochemical weapon. Spider venom-based chemical compound discovery provides an opportunity to obtain natural compounds with various pharmacological properties [22,23]. To the best of our knowledge, as a novel member of the genus *Alopecosa*, the bioactive peptides of the *A. nagpag* spider venom of have not been reported [24]. In this study, we identified a novel denfesin-like antiviral peptide, An1a, from the venom of the *A. nagpag* spider which targets the NS2B–NS3 protease of both DENV2 and ZIKV. Our data have demonstrated that An1a may have great potential to be optimized as a broad-spectrum anti-flavivirus compound. Considering that An1a may have some drawbacks for further clinical use, the in vivo antiviral effects of An1a with or without type I interferon co-administration should be evaluated in animal models. In addition, developing natural peptide-derived analogs with improved pharmacological properties and reduced side effects is also an option [10,31,32].

Despite their differences in pathogeneses, DENV and ZIKV are representative, emerging mosquito-borne flaviviruses with similar replication cycles which infect millions of people annually [33]. The non-structural proteins of flaviviruses are important for virus replication and assembly [1]. It has been established that NS2B–NS3 forms the viral protease and NS3 alone serves as the dsRNA helicase [34]. Recently, the NS2B–NS3 protease became an attractive antiviral target, and many of its inhibitors have been reported as potential antivirals [16,20,35]. Protease inhibitors are ubiquitously distributed in living organisms such as plants, animals, fungi, and bacteria, and these inhibitors can target proteinases through different mechanisms, resulting in either complete or partial inhibition. A large number of studies have suggested that protease inhibitors have promising therapeutic uses in the treatment of many diseases [36,37]. As reported previously, bromocriptine has been confirmed to inhibit ZIKV protease activity and viral replication by interacting with H51 and S135 in the ZIKV NS2B–NS3 protease [21]. Our results have shown that bromocriptine also inhibits the DENV2 NS2B–NS3 protease and its replication. This might well be due to the high sequence similarity of the NS2B–NS3 protease between ZIKV and DENV2, especially the active site residues of the proteolytic cavity involving H51 and S135 (Figure S2). It is reasonable to speculate that An1a may also interact with the active site of both the DENV2 and ZIKV NS2B–NS3 proteases, and this potential interaction needs exploration in the future.

In recent years, the threats of mosquito-transmitted DENV and ZIKV to public health have been increasing worldwide. The development of specific drugs for these emerging Flaviviruses infections is lagging. To meet the growing demand for peptide antiviral development, animal venom has become an important resource of novel antivirals [8,9,10]. In this study, we presented a novel defensive peptide, An1a, identified from the venom of the *A. nagpag* spider with antiviral activities against DENV2 and ZIKV infection. Despite the first identified antiviral peptide in the venom of the *A. nagpag* spider, the other components of the venom still remain largely unknown. Considering the complexity of *A. nagpag* spider venom, much more effort should be undertaken to understand its bioactive peptides. Moreover, this study has highlighted the existence of potential therapeutic anti-flaviviruses peptides in spider venoms.

## 4. Materials and Methods 

### 4.1. Venom Collection

Adult *A. nagpag* (female, *n* = 400) spiders were captured from Shangri-La County in Yunnan province, China. Venom was collected after the chelicerae were stimulated by using a 3 voltage alternating current, as described in our previous study [38]. Following collection, venom was lyophilized and immediately stored at −80 °C until further use.

### 4.2. Toxin Purification and the Sequence Determination of An1a

The spider venom was loaded on a Sephadex G-75 (2.6 × 100 cm; Superfine, Amersham Biosciences, GE Health, Chicago, IL, USA) gel filtration column pre-equilibrated with 0.05 M PBS, pH 7.2. Proteins were eluted at a flow rate of 0.3 mL/min, and fractions were collected every 3 mL. Peptidic fractions with antiviral activities were further purified by using C_18_ reverse-phase high performance liquid chromatography (RP-HPLC) (Unisil C_18_ column, 5 μm particle size, 10 × 250 mm, Suzhou, China). Elution was performed at a flow rate of 1.5 mL/min with the indicated gradients of acetonitrile in 0.1% (*v*/*v*) trifluoroacetic acid (TFA) in water. The complete amino acid sequence was determined by Edman degradation in an Applied ProciseTM 491-A protein sequencer (Shimadzu Corporation, Kyoto, Japan).

The spider venom gland cDNA library was prepared as previously described in our study [38]. Briefly, the total RNA was extracted from the venom glands of 2 female spiders using TRIzol (Life Technologies Ltd.) and was used to prepare cDNA using a SMART™ PCR cDNA synthesis kit (Clontech, Palo Alto, CA, USA). The cDNAs were cloned into a pGEM^®^-T Easy vector (Promega, Madison, WI, USA), and the colonies were random selected and amplified by colony PCR using the M13 primers (M13 Forward 5′-TGTAAAACGACGGCCAGT-3′, M13 reverse 5′CAGGAAACAGCTATGACC-3′). The PCR products which size >350 bp were chosen and sequenced on an ABI PRISM 377 DNA sequencer (Applied Biosystems, Waltham, MA, USA). The cDNA sequences were then translated into an amino acid sequence in the correct open reading frame. After obtaining the encoding cDNA sequence and full-length precursor of An1a by a sequence analysis, the sequence of An1a was also subjected to the signal peptide prediction tool (Online SignalP 5.0 Server, http://www.cbs.dtu.dk/services/SignalP/) to verify the signal peptide and mature peptide. 

### 4.3. Recombinant Expression of An1a

DNA sequences encoding An1a were synthesized and cloned into a pET-32a vector (TsingKe Biotech, Co., Ltd., Beijing, China). The designed cleavage site -ENLYFQ-, which is susceptible to the TEV protease, was inserted between the His-Tag and 5′ upstream of the An1a coding sequence. The An1a/pET-32a construct was transformed into the *Escherichia coli* strain BL-21 (DE3) for expression [21]. After hydrolysis by the TEV protease, recombinant An1a was purified through one step of Sephadex G-50 (2.6 × 100 cm; Superfine, Amersham Biosciences, GE Health, Chicago, IL, USA) gel filtration for preliminary fractionation and one step of C_18_ RP-HPLC, as described above, for final purification. The purity of recombinant An1a was confirmed to be greater than 98% before use.

### 4.4. Cells and Viruses

A549 cells, Vero cells and HUVEC cells were obtained from Kunming Cell Bank, Kunming Institute of Zoology, Chinese Academy of Science. A549 cells and Vero cells were cultured in a DMEM/F12 medium (Gibco, Waltham, MA, USA) supplemented with 10% fetal bovine serum (FBS), 100 U/mL of penicillin, and 100 μg/mL of streptomycin in 5% CO_2_ at 37 °C. HUVEC cells were cultured in an RPMI1640 medium (Gibco, Waltham, MA, USA) with same supplements. ZIKV SZ01 and DENV2 were kindly provided by Professor Cheng-feng Qin (Beijing Institute of Microbiology and Epidemiology).

### 4.5. In Vitro Virus Infection Assays and Antiviral Activity Measurement

For initial antiviral activity test, the lyophilized peptide fractions (100 μg/mL), bovine serum albumin (BSA, 100 μg/mL), and amodiaquine (AQ, 5 μM, A2799, Sigma, Burlington, MA, USA) were added to Vero cells post 1 MOI DENV2 infection in 96-well plates. After 24 h, the supernatants were collected for analysis. To further test the antiviral activity of An1a, cells were infected with DENV2 or ZIKV at 1 MOI for 24 h with or without An1a and bromocriptine mesylate (ab120574, Abcam, Cambridge, MA, USA) administration. Cells were then lysed in a TRIzol reagent for RNA isolation or fixed in 4% paraformaldehyde for immunofluorescence staining. The supernatants of the cells were applied for plaque forming assay, as previously described [6].

For the real-time qPCR analysis, total RNA was isolated from cells, and cDNA was reverse transcribed by using M-MLV reverse transcriptase (Promega, Madison, WI, USA). Real-time qRT-PCR was performed on the StepOnePlus Real-Time PCR Systems (Thermo, Waltham, MA, USA). Primer sequences are listed in Supplementary Table S1.

### 4.6. Immunofluorescence Staining and Microscopy

For immunostaining, cells were fixed in 4% paraformaldehyde for 15 min at room temperature and were then permeabilized with 0.3% Triton X-100 (T0694, Amresco, Houston, TX, USA) for 20 min, washed with PBS, and blocked in 2% BSA for 1 h. Cells were stained with an anti-DENV (sc-70959, Santa Cruz Biotechnology, Inc., Dallas, TX, USA) antibody at a 1:100 dilution overnight at 4 °C. Cells were washed, stained with fluorescence-conjugated secondary antibody with or without FITC-labeled Phalloidin (40735ES75, Yeasen, Shanghai, China) for 0.5 h at room temperature and mounted with ProLong Gold Antifade Reagent with DAPI (8961S, CST, Danvers, MA, USA). Immunostaining was detected by the Olympus FluoView 1000 confocal microscope (Olympus, Tokyo, Japan).

### 4.7. Recombinant Protease Expression, Protease Inhibition and the Mechanism of Inhibition

The DNA encoding the DENV2 and ZIKV NS2B–NS3 proteases were synthesized (TsingKe Biotech, Co., Ltd., Beijing, China), and two amino acids were replaced in order to avoid autoproteolytic cleavage, as previously reported [30]. The construct codes of NS2B and NS3 were connected by a Gly4–Ser–Gly4 linker. Both of the DNA were ligated into pET-28b, and the plasmids were transformed into *E. coli* strain BL-21(DE3) for expression, and the recombinant DENV2 and ZIKV NS2B–NS3 proteases were purified as previously reported [21,30].

To test if An1a inhibited protease activity, assays were conducted in 96-well black micro plates utilizing Bz–Nle–Lys–Lys–Arg–AMC (GL Biochem Ltd., Shanghai, China) as a substrate [21]. Monitoring was initiated, and the fluorescence of each well was recorded every 30 s using an excitation of 360 nm and emission of 460 nm on a FlexStation microplate reader (Molecular Devices, Sunnyvale, CA). Results were determined as relative fluorescence units (RFU). Curves were generated using the GraphPad Prism 6 software (Version 6.01, GraphPad Software, Inc., San Diege, CA, 2012).

### 4.8. Assays of Hemolysis and Cytotoxicity

A hemolysis assay was undertaken as previously reported [31]. Maximum hemolysis was determined by adding 1% Triton X-100 to a sample of cells. The hemolysis of testing samples was calculated as the percentage of Triton X-100. Cell viability was evaluated by conventional 3-(4, 5-dimethyl-2-thiazolyl)-2, 5-diphenyl-2H-tetrazolium bromide (MTT) reduction assays. After a 24 h treatment of the testing sample, 20 μL of MTT (5 mg/mL) was added to each well. The MTT solution was then removed, and 200 μL dimethyl sulfoxide (DMSO) was added to solubilize the MTT-formazan crystals in living cells. The absorbance at 570 nm of the resulting solution was measured. 

### 4.9. Statistical Analysis

Data are given as mean ± SEM. A statistical analysis was performed using a two-tailed Student t-test, and log-rank tests. A *p*-value ≤ 0.05 was considered significant.

## Supplementary Materials

The following are available online at https://www.mdpi.com/2072-6651/11/10/584/s1, Figure S1: The representative images showing the sequenced PCR products length from selected clones, Figure S2: The cytotoxicity and hemolytic activity of An1a in vitro, Figure S3: Multiple sequence alignment of the NS2B–NS3 proteases of flaviviruses, Figure S4. Purification of the crude spider venom, Table S1: Primers used in this study.
