# Supplementary material for: An Antiviral Peptide from Alopecosa nagpag Spider Targets NS2B–NS3 Protease of Flaviviruses
Source: Toxins (Basel). 2019 Oct 10;11(10):584. doi: 10.3390/toxins11100584 (PMC6832551; doi:10.3390/toxins11100584)
Supplement: Supplementary file 1 [file toxins-11-00584-s001.pdf]

Supplementary Materials: An Antiviral Peptide from *Alopecosa nagpag* Spider Targets NS2B–NS3 Protease of Flaviviruses

Mengyao Ji, Tengyu Zhu, Meichen Xing, Ning Luan, James Mwangi, Xiuwen Yan, Guoxiang Mo, Mingqiang Rong, Bowen Li, Ren Lai and Lin Jin

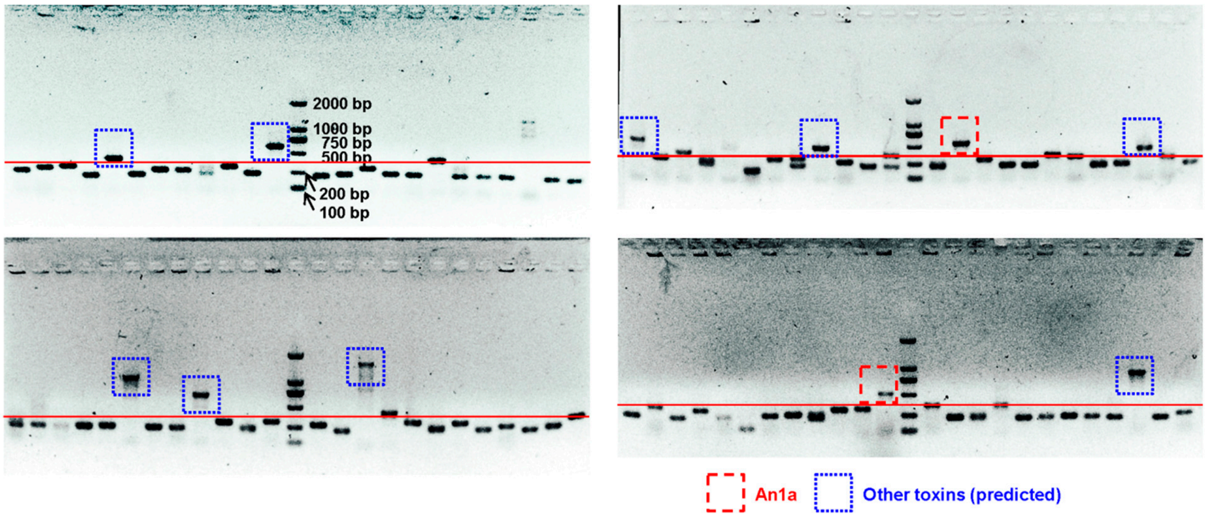

Figure S1. The representative images showing the sequenced PCR product length from selected clones. Bands were boxed with different color as indicated.

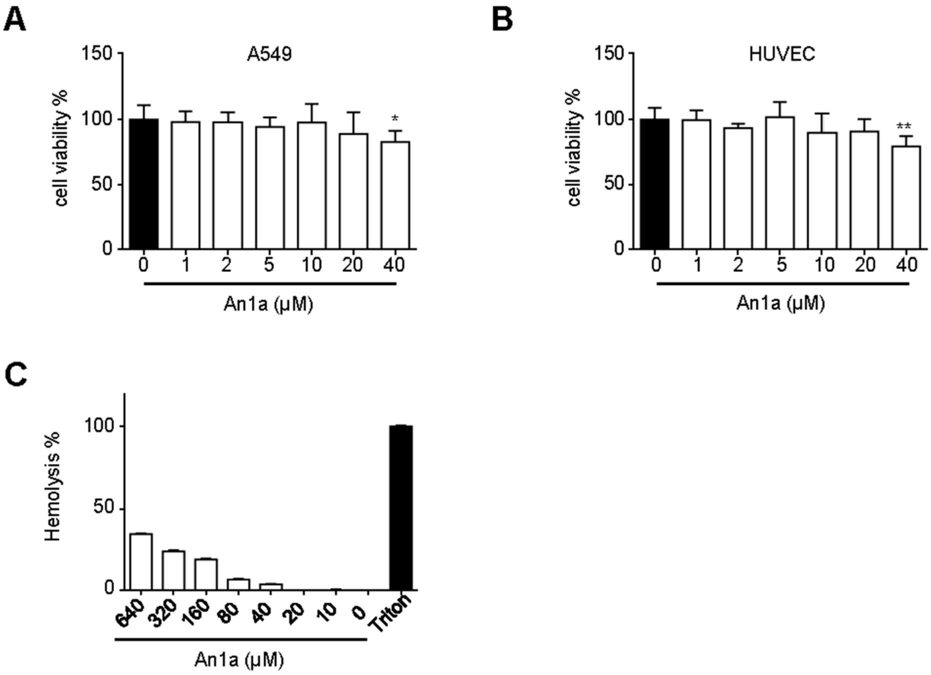

Figure S2. The cytotoxicity and hemolytic activity of An1a in vitro. (**A**) The cytotoxicity of An1a on HUVECs and (**B**) A549 cells. (**C**) The hemolytic activity of An1a on human red blood cells. The Triton-X 100 treatment was set as 100%. Data represent at least two independent experiments and are presented as mean ± SEM. * $p < 0.05$, ** $p < 0.01$.

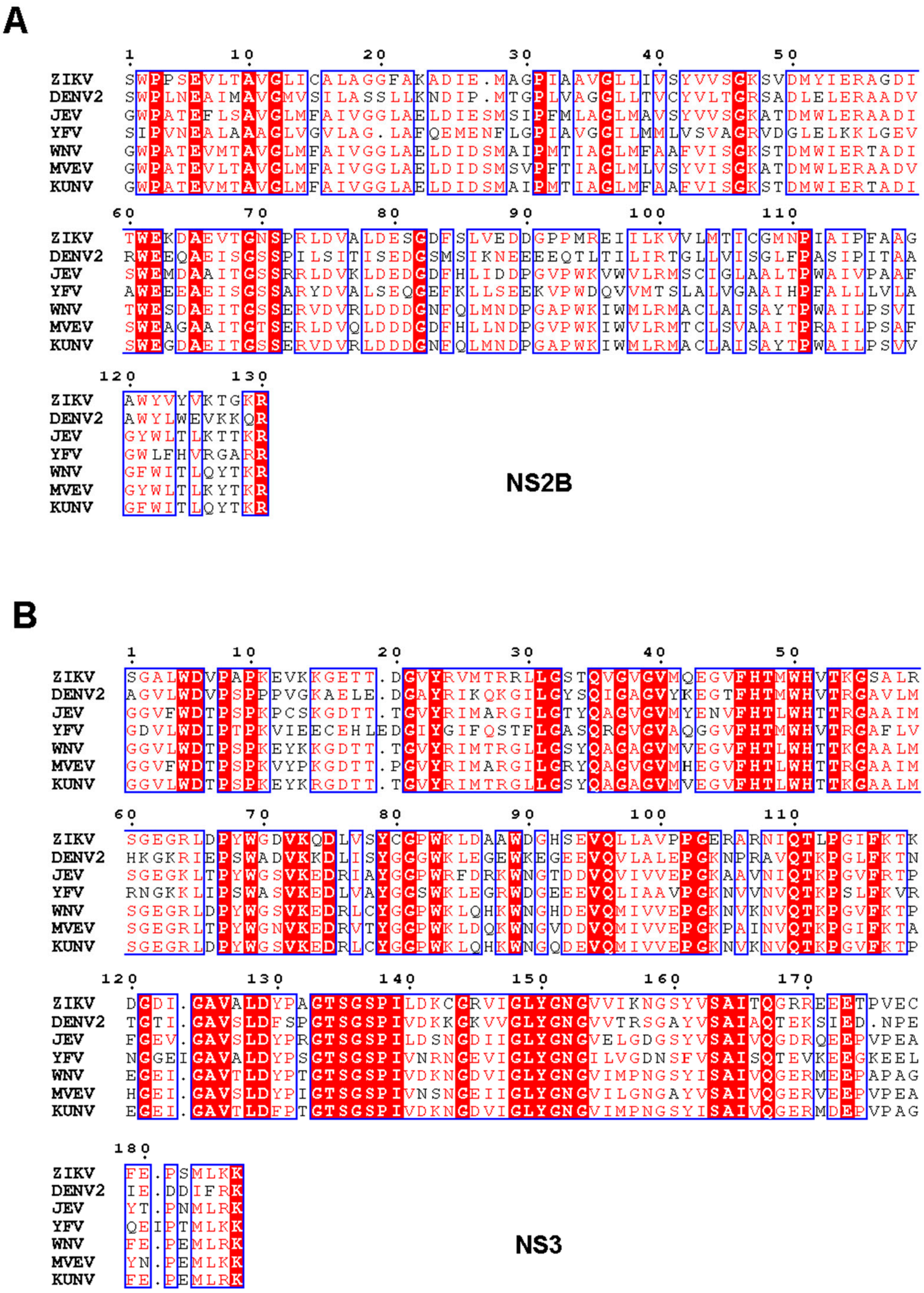

Figure S3. Multiple sequence alignment of the NS2B–NS3 proteases of flavivireses. (**A**) The sequences of NS2B protein. (**B**) The sequences of NS3 protein.

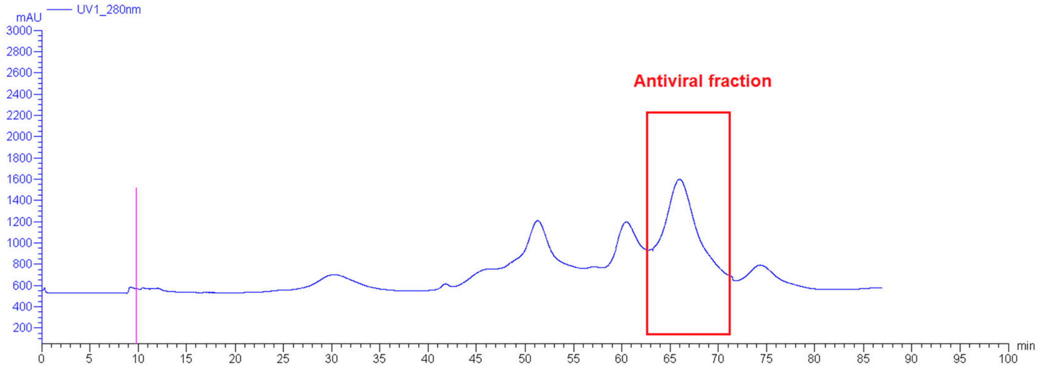

Figure S4. Purification of the crude spider venom. The crude venom was loaded on a Sephadex G-75 (26 × 100 cm; Superfine, Amersham Biosciences) gel filtration column on an AKTA Explorer fast protein liquid chromatography system (GE Healthcare) with 0.02 M PBS, pH 7.0, and eluted at a flow rate of 1 mL/min. The absorbance of eluted peaks was monitored at 280 nm.

Table S1. Real time qPCR primer sequences.

Gene	5′ Primer	3′ Primer
Zikv	GACGCCAGAGTTTGTTCAGA	TGGCTTCCTGGAATCTCTCT
Denv2	CAGGCTATGGCACYGTCACGA	CCATYTGCAGCARCACCATCTC
hHprt	GCTATAAATTCTTTGCTGACCTGCTG	AATTACTTTTATGTCCCCTGTTGACTGG
mHprt	CTCATGGACTGATTATGGACAGGAC	GCAGGTCAGCAAAGAACTTATAGCC
